# Effects of Exogenous Galanin on Neuropathic Pain State and Change of Galanin and Its Receptors in DRG and SDH after Sciatic Nerve-Pinch Injury in Rat

**DOI:** 10.1371/journal.pone.0037621

**Published:** 2012-05-18

**Authors:** Xiaofeng Xu, Xiangdong Yang, Ping Zhang, Xiuying Chen, Huaxiang Liu, Zhenzhong Li

**Affiliations:** 1 Department of Anatomy, Shandong University School of Medicine, Jinan, China; 2 Department of Nephrology, Shandong University Qilu Hospital, Jinan, China; 3 Department of Electroencephalography, Shandong University Affiliated Shandong Provincial Hospital, Jinan, China; 4 Department of Rheumatology, Shandong University Qilu Hospital, Jinan, China; Charité University Medicine Berlin, Germany

## Abstract

A large number of neuroanatomical, neurophysiologic, and neurochemical mechanisms are thought to contribute to the development and maintenance of neuropathic pain. However, mechanisms responsible for neuropathic pain have not been completely delineated. It has been demonstrated that neuropeptide galanin (Gal) is upregulated after injury in the dorsal root ganglion (DRG) and spinal dorsal horn (SDH) where it plays a predominantly antinociceptive role. In the present study, sciatic nerve-pinch injury rat model was used to determine the effects of exogenous Gal on the expression of the Gal and its receptors (GalR1, GalR2) in DRG and SDH, the alterations of pain behavior, nerve conduction velocity (NCV) and morphology of sciatic nerve. The results showed that exogenous Gal had antinociceptive effects in this nerve-pinch injury induced neuropathic pain animal model. It is very interesting that Gal, GalR1 and GalR2 change their expression greatly in DRG and SDH after nerve injury and intrathecal injection of exougenous Gal. Morphological investigation displays a serious damage after nerve-pinch injury and an amendatory regeneration after exogenous Gal treatment. These findings imply that Gal, via activation of GalR1 and/or GalR2, may have neuroprotective effects in reducing neuropathic pain behaviors and improving nerve regeneration after nerve injury.

## Introduction

Damages to the nervous system are the primarily cause of neuropathic pain [1]. A large number of neuroanatomical, neurophysiologic, and neurochemical mechanisms are thought to contribute to the development and maintenance of neuropathic pain [2]. Neuropathic pain is a severe health problem for which there is a lack of effective therapy [3]. Even in present day pain therapy, neuropathic pain remains a challenge for clinicians to treat and a challenge for researchers to investigate [4]. Mechanisms responsible for neuropathic pain have not been completely delineated. Different animal models have been developed to mimic neuropathic pain. Sciatic nerve-pinch injury is a reliable animal model for evaluating pain behavior [5].

Dorsal root ganglion (DRG) is anatomically discrete peripheral nervous system (PNS) structure located on the dorsal root of each spinal nerve immediately adjacent to the spinal cord. Each ganglion contains the cells bodies of sensory neurons of pseudounipolar structure, the stem axons of which send processes peripherally into the corresponding spinal nerve and centrally into the spinal cord where they synapse on the neurons in the spinal cord and brainstem of central nervous system (CNS). DRG is recognized as one of the organs which are damaged in peripheral sensory neuropathic pain states [6]. It has been suggested that the spinal cord is a site of amplified sensory processing in neuropathic pain states [7], [8]. Spinal dorsal horn (SDH) is important in mediating nociceptive signaling [9]–[12]. It has been well recognized that nerve lesion induces change of gene and protein expression of neuropeptides and receptors in DRG and SDH which correspond to induced neuropathic pain [13], [14].

Neurotrophic peptide galanin (Gal) has been shown to be effective in preventing or reversing neuropathic pain in animal models [15], [16]. Gal, a 29-amino-acid neuropeptide in most species (30-amino-acid in human), is widely expressed in both the PNS and CNS and is involved in many diverse biological functions including nociception, developmental and trophic effects [17]–[21]. Gal is present in a small population of DRG neurons and is thought to play only a minor role in nociception under normal conditions. However, there is a substantial data set that demonstrates Gal is increased after injury in the DRG and spinal cord where it plays a predominantly antinociceptive role in addition to being neuroprotective and pro-regenerative [22]. These data led us to speculate that Gal might play a paregoric and trophic role in nerve injury-induced neuropathic pain model. Hence, it is important to discover whether exogenous Gal improves pain behaviour, nerve conduction velocity (NCV) and axonal morphology after nerve injury. In the present study, sciatic nerve-pinch injury rat model was used to determine the effects of exogenous Gal on the expression of the Gal and its receptors (GalR1, GalR2) in DRG and SDH, the alterations of pain behavior, NCV and morphology of sciatic nerve.

## Materials and Methods

### Ethics Statement

All animals were cared for in compliance with the Guide for the Care and Use of Laboratory Animals (revised 1996; http://www.nap.edu). All procedures described herein were reviewed by and had prior approval by the Ethical Committee for Animal Experimentation of the Shandong University. All surgery was performed under anesthesia, and all efforts were made to minimize suffering.

### Animal and catheter implantation

All preparations utilized male rats (300 g∼350 g) taken from the breeding colony of Wistar rats maintained in the Experimental Animal Center at Shandong University of China. All animals were housed in plastic cages with a normal light-dark cycle keeping room temperature 20°C∼25°C and allowed free access to chow and water. The rats were anesthetized with of 10% chloral hydrate (300 mg/kg) intraperitoneally (i.p.) before catheter implantation. A sterile polyethylene catheter (PE-10, 15.0 cm length) (Instech Laboratories Incorporation, Plymouth Meeting, PA, USA) was inserted into the subarachnoid space through an incision in the gap between the lumbar (L)6 and sacral (S)1 vertebra. The tip of the catheter was implanted between L4 and L5 DRG level. Three days after implantation, the correct catheter placement was confirmed by intrathecally injecting 10 µl 2% lidocaine. Only the rat showing a reversible paralysis of the hind paws within 30 seconds following the lidocaine injection were considered to be catheterized successfully.

### Sciatic nerve-pinch injury pain animal model

After the baseline pain behavior tests were finished, the successfully catheterized rats were anesthetized with 10% chloral hydrate. Bilateral sciatic nerve were exposed and pinched for 3 seconds with a microsurgery clamp (0.3 mm tip) at the point where the nerve came across the adductor brevis muscle. The surgical procedures of the sham-operated animals were same as the operated animals except nerve pinch.

### Animal groups and intrathecal drug administration

Sixty-three rats with successful catheter implantation were randomly divided into 3 groups (21 rats in each group). Group 1: Animals with sciatic nerve-pinch injury were treated with vehicle solution (i.t.). Group 2: Animals with sciatic nerve-pinch injury were treated with Gal (3 µg/day, i.t.) (Tocris Bioscience, Bristol, UK). Group 3: The sham-operated control animals were treated with vehicle solution (i.t.). At the day 8, 16, and 24 (each time point 7 rats in each group) after sciatic nerve-pinch injury, animals were processed for NCV measurement after anesthesia with 10% chloral hydrate. After that, the L4-5 DRG and the corresponding SDH and sciatic nerve explants (2 mm distal to the pinch injury point) were surgically harvested for different experimental examination.

Gal was dissolved in artificial cerebrospinal fluid (ACSF) at 0.3 µg/µl. The composition of ACSF (pH7.4) is as follows (mmol/L): NaCl, 138.6; KCl, 3.35; CaCl_2_·2H_2_O, 1.26; MgCl_2_·6H_2_O, 1.16; NaH_2_PO_4_·2H_2_O, 0.58; NaHCO_3_, 21.0; glucose, 10.0 [23].

### Evaluation of mechanical and thermal hyperalgesia

The threshold responses to mechanical and thermal stimuli were tested daily on both hind paws with Von Frey filaments (BME-403, Chinese academy of medical sciences institute of biomedical engineering) and a plantar analgesia tester (BME-400C, Chinese academy of medical sciences institute of biomedical engineering), respectively.

Mechanical hyperalgesia was determined by 50% withdrawal thresholds to mechanical stimuli applying to the hind paws. All rats involved in this test were acclimated in a clear plastic cage with a wire mesh bottom individually for 15 minutes. A series of filaments (0.1, 0.5, 1.0, 3.0, 3.8, 5.0, 8.2, and 14.6 g), starting with 3.0 g, were used serially to stimulate the central region of the plantar surface of the rat's hind paws in ascending or descending order of stiffness depending on the foot withdrawal response of the rats. The filament was applied with a pressure that was just sufficient to bend the filament for 5 seconds. A brisk withdrawal of the hind paw was recorded as a positive response. The interval between the two stimuli was at least 5 seconds. Both hind paws were tested twice for average. The score was cut off at a force of 14.6 g to prevent the hind paw from injury. The 50% threshold to mechanical stimuli was interpolated according to the sequence of positive and negative scores.

Thermal hyperalgesia was determined as the withdrawal latency to a noxious radiant heat stimulus. Each rat was placed in a transparent plastic chamber (15×20×30 cm) on a thin glass platform maintained at 30°C. All animals involved in this test were acclimated to the environment for 15 minutes before testing. A mobile radiant heat lamp generated by a high intensity light bulb (10 W) was then positioned directed underneath the targeted hind paw. The mean withdrawal latency was determined by 4 tests (after excluding the minimal and maximal values). A cutoff of 20 seconds was used to avoid tissue injury.

### NCV determination

Both motor NCV (MNCV) and sensory NCV (SNCV) of the rat sciatic nerve was determined by a Keypoint electromyography (EMG)/evoked potentials (EP) systems (Dantec, Copenhagen, Denmark). Briefly, after anesthesia with 10% chloral hydrate, rat was placed on a heating mat to maintained rectal temperature at 38°C. Sciatic nerve conduction was performed by stimulating proximally at the sciatic notch which is almost 0.7 cm above the pinch point and distally at he Achilles tendon with bipolar electrodes. The latencies of M-wave (compound muscle action potential) and H-reflex were recorded via a needle electrode from the second interosseous muscle of the hind paw. The MNCV and SNCV were calculated by subtracting the distal and proximal latencies, and the result was divided into the distance between the two stimulating points. The velocities were recorded as meters per second.

### Real time-PCR analysis of mRNAs for Gal, GalR1, and GalR2

At the designed experimental time point, the L4-5 DRG and the corresponding SDH of the rats were surgically harvested. The mRNA levels of Gal, GalR1, and GalR2 in L4-5 DRG and the corresponding SDH were analyzed by real time-PCR. The expression of glyceraldehyde-3-phosphate dehydrogenase (GAPDH) mRNA was also determined as an internal control. Total RNA from DRG and SDH tissues was isolated by TRIzol (Invitrogen, Grand Island, NY). cDNA was synthesized using cDNA synthesis kit (Fermentas, EU) according to the manufacturer's instructions. The synthetic oligonucleotide primer sequences for Gal, GalR1, GalR2 and GAPDH were as follows: Gal 5′- TGC AAC CCT GTC AGC CAC TC -3′ (coding sense) and 5′- TGT CGC TAA ATG ATC TGT GGT TGT C -3′ (coding antisense). GalR1 5′- AGG CTT ACG TGG TGT GCA CTT TC -3′ (coding sense) and 5′- GCC ATG ATA TGC CAA ATA CCA CAA -3′ (coding antisense). GalR2 5′- CAT CGT GGC GGT GCT TTT -3′ (coding sense) and 5′- AGC GGG AAG CGA CCA AAC -3′ (coding antisense). GAPDH 5′- GGC ACA GTC AAG GCT GAG AAT G -3′ (coding sense) and 5′- ATG GTG GTG AAG ACG CCA GTA -3′ (coding antisense).

The amplification was conducted in a volume of 25 µl containing 12.5 µl of Maxima SYBR Green qPCR Maxter Mix (2×) (Fermentas, EU), with 0.3 µmol/L forward and reverse primers, 2 µl cDNA. Real time RT-PCR was carried out using the eppendorf Realplex PCR system (Eppendorf, Hamburg, Germany). PCR cycle conditions were as follows: activated at 95°C for 10 min, followed by amplification and quantification 40 cycles at 95°C for 15 s, 60°C for 30 s and 72°C for 30 s. A comparative cycle of threshold fluorescence (Ct) method was used and the relative transcript amount of the target gene was normalized to that of GAPDH using the 2^−ΔΔCt^ method based on a previous study [24]. The final results of real time-PCR were expressed as the ratio of mRNA of control.

### Western blot assay of Gal, GalR1, and GalR2

At the designed experimental time point, the L4-5 DRG and the corresponding SDH of the rats were surgically harvested. The levels of Gal, GalR1, and GalR2 in L4-5 DRG and the corresponding SDH were analyzed by Western blot assay. The expression of β-actin was also determined as an internal control. The DRG and SDH tissues were homogenized in 10 mmol/L Tris homogenization buffer (pH 7.4) with protease inhibitors. The samples were centrifuged at 10,000 g for 10 minutes and the supernatant collected for Western blot assay. After determining the protein concentrations of the supernatants (BCA method, standard: BSA), 50 µg protein of each sample was loaded onto the 10% SDS gel, separated by electrophoresis and transferred to nitrocellulose membrane. The membranes were blocked in blocking buffer (5% nonfat milk) for 2 hours at room temperature, and then were incubated with goat anti-Gal polyclonal IgG (1∶500, Santa Cruz Biotechnology), goat anti-GalR1 polyclonal IgG (1∶500, Santa Cruz Biotechnology), goat anti-GalR2 polyclonal IgG (1∶500, Santa Cruz Biotechnology) or mouse anti-β-actin monoclonal IgG (1∶1000, Santa Cruz Biotechnology) overnight at 4°C. After being washed three times for 10 minutes with washing solution, the membranes were incubated with rabbit anti-goat IgG-HRP (1∶4000, Santa Cruz Biotechnology) or goat anti-mouse IgG-HRP (1∶4000, Santa Cruz Biotechnology). The immunoreactive bands were visualized by an ECL Western blotting detection kit (Billerica) on light sensitive film. The films were scanned and the images were analyzed quantitatively by using an ImagJ 1.39 u image analysis software. The protein levels of Gal, GalR1, and GalR2 were expressed as the ratio of control.

### Morphological observation

At the conclusion of the experiments, sciatic nerve explants (2 mm distal to the pinch injury point) were fixed in a 4%paraformaldehyde/3%glutaraldehyde solution for 30 minutes at 4°C. The samples were postfixed in 1% osmium tetroxide in 0.1 mol/L phosphate buffer (PB) for 2 hours at 4°C and embedded in Epon for 72 hours at 60°C. For toluidine blue staining, transverse semi-thin sections (1 µm) of sciatic nerve were cut with an ultramicrotome (Leica EM UC6), stained with 0.5% toluidine blue and observed under a light microscope (Nikon Eclipse 80i, Japan). For ultrastructural observation, ultra-thin sections (70 nm) of sciatic nerve were processed for electron microscopy and negatively stained with uranyl acetate and lead citrate and examined with a JEOL-1400 transmission electron microscope (TEM).

### Statistical analysis

Data are expressed as mean ± SD. The normal distribution of all data was verified. If normality test is fail, the data were analyzed with non-parametric test. If normality test is pass, statistical analysis was evaluated with SPSS software by one-way ANOVA followed by the Student-Newman-Keuls test for significance to compare the differences among various groups. *t* test was used for analysis of two independent samples. Significance was determined as *P*<0.05.

## Results

### Effects of exogenous Gal on pain behaviour after sciatic nerve-pinch injury

**Figure 1:**
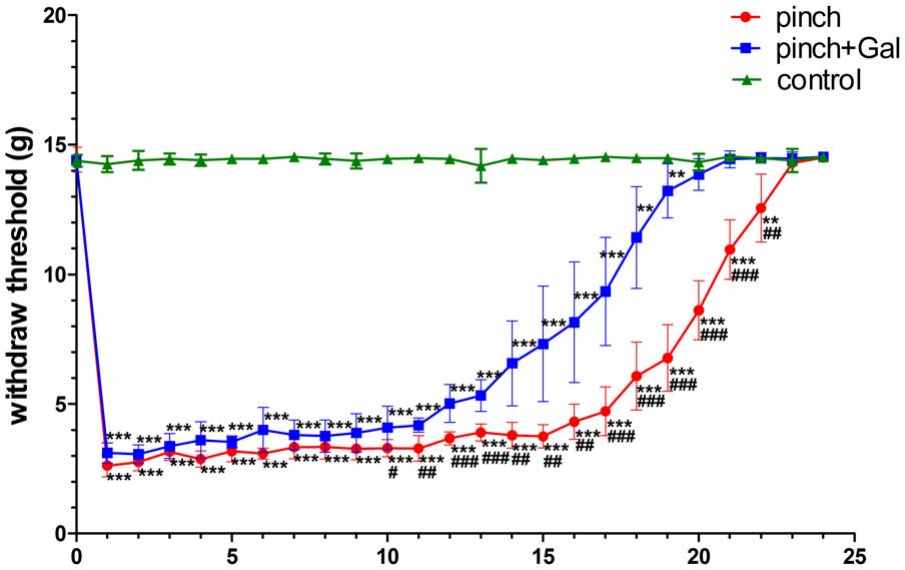
The mechanical threshold alterations with different treatment from day 0 to day 24. The data are presented as means ± SD (n = 7). *P<0.05 *vs*. control, **P<0.01 *vs*. control, ***P<0.001 *vs*. control, ^#^ *P<*0.05 *vs*. pinch + Gal group, ^##^ *P<*0.01 *vs*. pinch + Gal group, ^###^ *P<*0.001 *vs*. pinch + Gal group.

**Figure 2:**
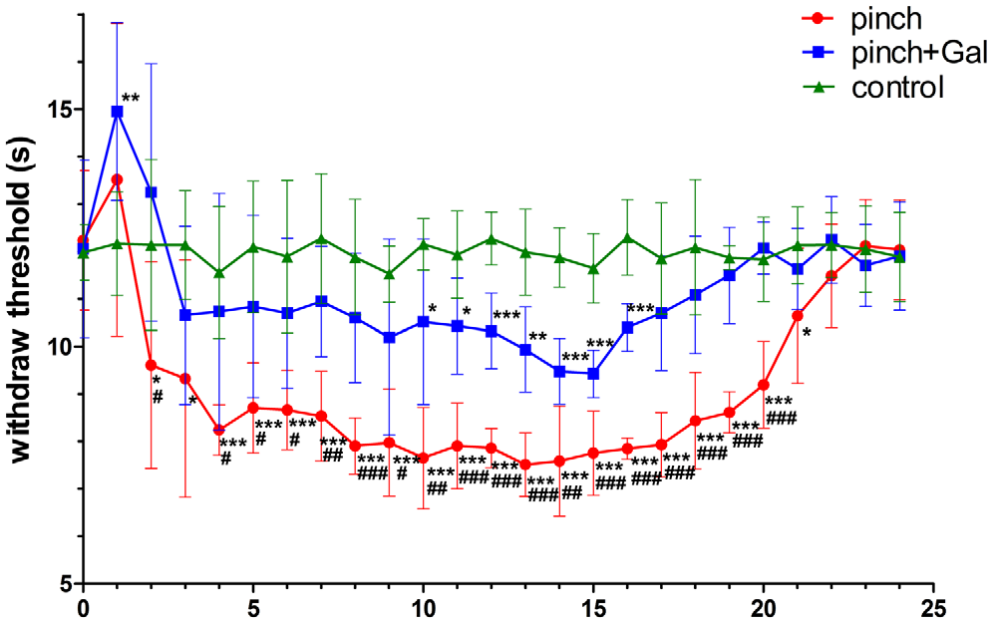
The thermal threshold alterations with different treatment from day 0 to day 24. The data are presented as means ± SD (n = 7). *P<0.05 *vs*. control, **P<0.01 *vs*. control, ***P<0.001 *vs*. control, ^#^ *P<*0.05 *vs*. pinch + Gal group, ^##^ *P<*0.01 *vs*. pinch + Gal group, ^###^ *P<*0.001 *vs*. pinch + Gal group.

Sciatic nerve-pinch injury caused mechanical and thermal hyperalgesia. The mechanical threshold decreased significantly after sciatic nerve-pinch injury from day 1 to day 22. The mechanical threshold recovered to normal baseline at day 23 after sciatic nerve-pinch injury (Fig. 1). The thermal threshold decreased significantly after sciatic nerve-pinch injury from day 2 to day 21. The thermal threshold recovered to normal baseline at day 22 after sciatic nerve-pinch injury (Fig. 2). Administration of Gal for sciatic nerve-pinch injured rats could substantially relieve pain and shorten recovery time of pain behavior. Gal administration increased mechanical threshold significantly after sciatic nerve-pinch injury from day 10 to day 22 as compared with that in sciatic nerve-pinched animals without Gal treatment. The mechanical threshold recovered to normal baseline at day 20 after sciatic nerve-pinch injury with Gal treatment (Fig. 1). Gal administration increased thermal threshold significantly after sciatic nerve-pinch injury from day 4 to day 20 as compared with that in sciatic nerve-pinched animals without Gal treatment. The thermal threshold recovered to normal baseline at day 17 after sciatic nerve-pinch injury with Gal treatment (Fig. 2). The mechanical and thermal thresholds showed no significant difference between different experimental time in sham-operated control animals (Fig. 1, Fig. 2).

### Effects of exogenous Gal on NCV after sciatic nerve-pinch injury

**Figure 3:**
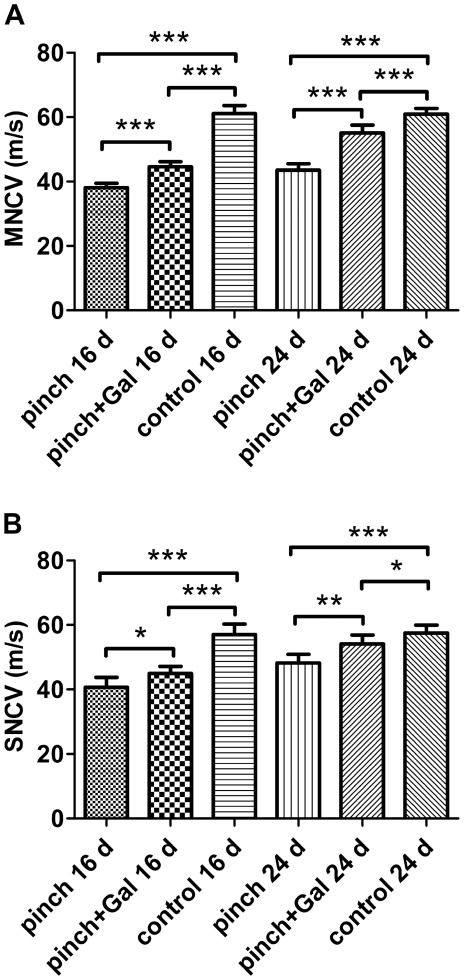
The NCV alterations at different experimental conditions. Panel A: MNCV decreased significantly after sciatic nerve-pinch injury at day 16 and 24 as compared with that in sham-operated control animals. Gal treatment improved MNCV significantly at the same experimental time point. Panel B: SNCV decreased significantly after sciatic nerve-pinch injury at day 16 and 24 as compared with that in sham-operated control animals. Gal treatment improved SNCV significantly at the same experimental time point. The data are presented as mean ± SD (n = 7). *P<0.05, **P<0.01, ***P<0.001.

Both MNCV and SNCV decreased significantly after sciatic nerve-pinch injury at day 16 and 24 (only few rats showing myoelectricity reaction at day 8, data not shown) as compared with that in sham-operated control animals. Gal administration improved MNCV and SNCV significantly after sciatic nerve-pinch injury at day 16 and 24 as compared with that in sciatic nerve-pinched animals without Gal treatment (Fig. 3).

### Expression of mRNAs for Gal, GalR1, and GalR2 in DRG and SDH

**Figure 4:**
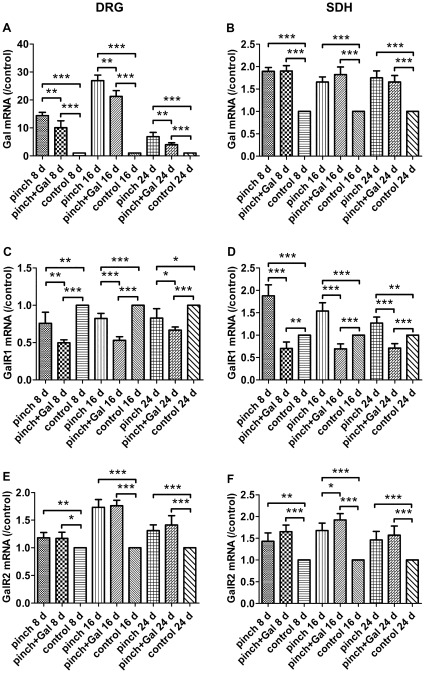
Expression of mRNAs for Gal, GalR1, and GalR2 in DRG and SDH. Panel A: Gal mRNA levels in DRG after sciatic nerve-pinch injury at day 8, 16, and 24 without or with Gal treatment. Panel B: Gal mRNA levels in SDH after sciatic nerve-pinch injury at day 8, 16, and 24 without or with Gal treatment. Panel C: GalR1 mRNA levels in DRG after sciatic nerve-pinch injury at day 8, 16, and 24 without or with Gal treatment. Panel D: GalR1 mRNA levels in SDH after sciatic nerve-pinch injury at day 8, 16, and 24 without or with Gal treatment. Panel E: GalR2 mRNA levels in DRG after sciatic nerve-pinch injury at day 8, 16, and 24 without or with Gal treatment. Panel F: GalR2 mRNA levels in SDH after sciatic nerve-pinch injury at day 8, 16, and 24 without or with Gal treatment. The mRNA level was expressed as the ratio of control. The data are presented as mean ± SD (n = 7). *P<0.05, **P<0.01, ***P<0.001.

Gal mRNA levels increased significantly in DRG and SDH after sciatic nerve-pinch injury at day 8, 16, and 24 as compared with that in sham-operated control animals. Gal administration decreased Gal mRNA levels in DRG, but not SDH, after sciatic nerve-pinch injury at day 8, 16, and 24 as compared with that in sciatic nerve-pinched animals without Gal treatment at the same experimental time point (Fig. 4A, B).

GalR1 mRNA levels decreased significantly in DRG after sciatic nerve-pinch injury at day 8, 16, and 24 as compared with that in sham-operated control animals. Gal administration decreased GalR1 mRNA levels in DRG after sciatic nerve-pinch injury at day 8, 16, and 24 as compared with that in sciatic nerve-pinched animals without Gal treatment at the same experimental time point (Fig. 4C). GalR1 mRNA levels increased significantly in SDH after sciatic nerve-pinch injury at day 8, 16, and 24 as compared with that in sham-operated control animals. Gal administration decreased GalR1 mRNA levels in SDH after sciatic nerve-pinch injury at day 8, 16, and 24 as compared with that in sciatic nerve-pinched animals without Gal treatment and in sham-operated control animals (Fig. 4D).

GalR2 mRNA levels increased significantly in DRG and SDH after sciatic nerve-pinch injury at day 8, 16, and 24 as compared with that in sham-operated control animals. Gal administration did not have significant effects on GalR2 mRNA expression in DRG and SDH after sciatic nerve-pinch injury as compared with that in sciatic nerve-pinched animals without Gal treatment at the same experimental time point (Fig. 4E, F).

### Expression of Gal, GalR1, and GalR2 in DRG and SDH

**Figure 5:**
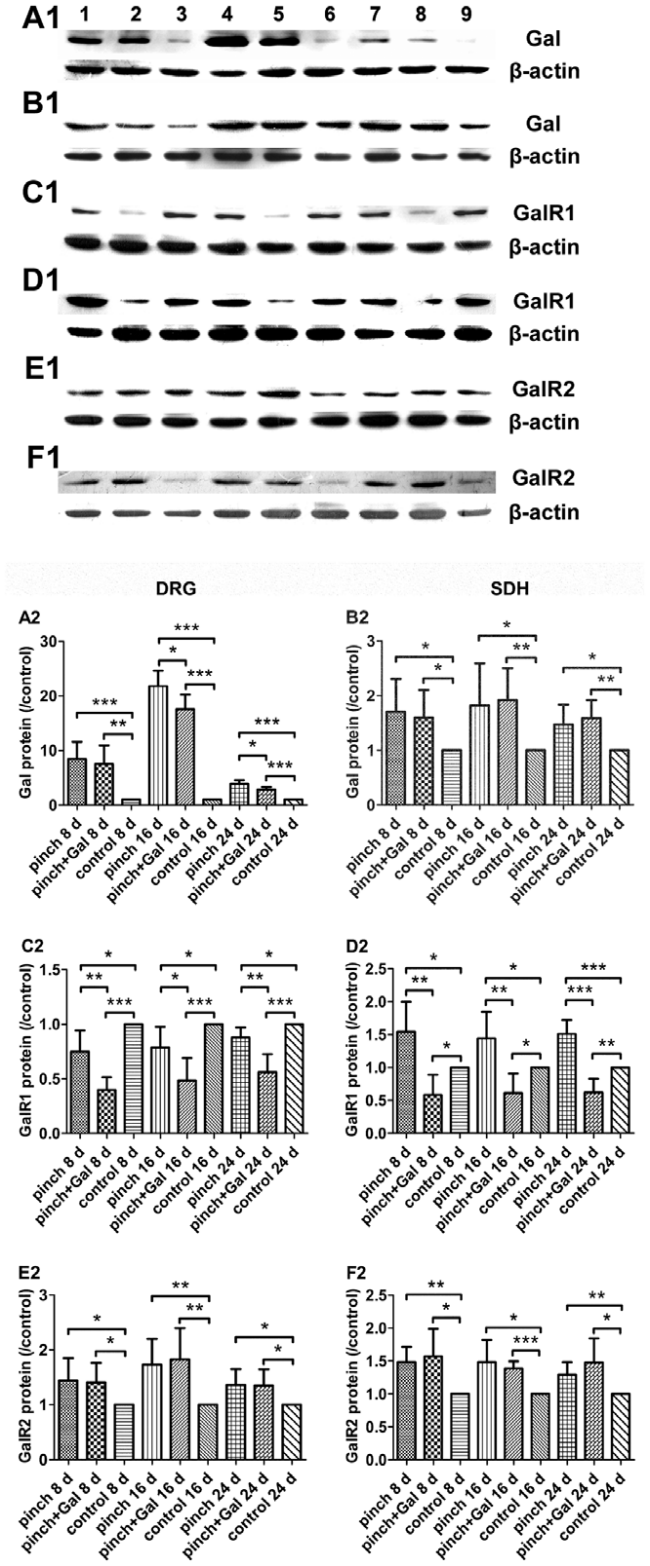
Expression of Gal, GalR1, and GalR2 in DRG and SDH. Panel A1, A2: Gal protein levels in DRG after sciatic nerve-pinch injury at day 8, 16, and 24 without or with Gal treatment. Panel B1, B2: Gal protein levels in SDH after sciatic nerve-pinch injury at day 8, 16, and 24 without or with Gal treatment. Panel C1, C2: GalR1 protein levels in DRG after sciatic nerve-pinch injury at day 8, 16, and 24 without or with Gal treatment. Panel D1, D2: GalR1 protein levels in SDH after sciatic nerve-pinch injury at day 8, 16, and 24 without or with Gal treatment. Panel E1, E2: GalR2 protein levels in DRG after sciatic nerve-pinch injury at day 8, 16, and 24 without or with Gal treatment. Panel F1, F2: GalR2 protein levels in SDH after sciatic nerve-pinch injury at day 8, 16, and 24 without or with Gal treatment. Lane 1: pinch 8 d; Lane 2: pinch + Gal 8 d; Lane 3: control 8 d; Lane 4: pinch 16 d; Lane 5: pinch + Gal 16 d; Lane 6: control 16 d; Lane 7: pinch 24 d; Lane 8: pinch + Gal 24 d; Lane 9: control 24 d. The protein level was expressed as the ratio of control. The data are presented as mean ± SD (n = 7). *P<0.05, **P<0.01, ***P<0.001.

Gal protein levels increased significantly in DRG after sciatic nerve-pinch injury at day 8, 16, and 24 as compared with that in sham-operated control animals. Gal administration decreased Gal protein levels in DRG after sciatic nerve-pinch injury at day 16 and 24, but not day 8, as compared with that in sciatic nerve-pinched animals without Gal treatment at the same experimental time point (Fig. 5A). Gal protein levels increased significantly in SDH after sciatic nerve-pinch injury at day 8, 16, and 24 as compared with that in sham-operated control animals. Gal administration did not have significant effects on Gal protein expression in SDH after sciatic nerve-pinch injury as compared with that in sciatic nerve-pinched animals without Gal treatment at the same experimental time point (Fig. 5B).

GalR1 protein levels decreased significantly in DRG after sciatic nerve-pinch injury at day 8, 16, and 24 as compared with that in sham-operated control animals. Gal administration decreased GalR1 protein levels in DRG after sciatic nerve-pinch injury at day 8, 16, and 24 as compared with that in sciatic nerve-pinched animals without Gal treatment at the same experimental time point (Fig. 5C). GalR1 protein levels increased significantly in SDH after sciatic nerve-pinch injury at day 8, 16, and 24 as compared with that in sham-operated control animals. Gal administration decreased GalR1 protein levels in SDH after sciatic nerve-pinch injury at day 8, 16, and 24 as compared with that in sciatic nerve-pinched animals without Gal treatment and in sham-operated control animals (Fig. 5D).

GalR2 protein levels increased significantly in DRG and SDH after sciatic nerve-pinch injury at day 8, 16, and 24 as compared with that in sham-operated control animals. Gal administration did not have significant effects on GalR2 protein expression in DRG and SDH after sciatic nerve-pinch injury as compared with that in sciatic nerve-pinched animals without Gal treatment at the same experimental time point (Fig. 5E, F).

### Morphological alterations of nerve fiber in sciatic nerve with toluidine blue staining

**Figure 6:**
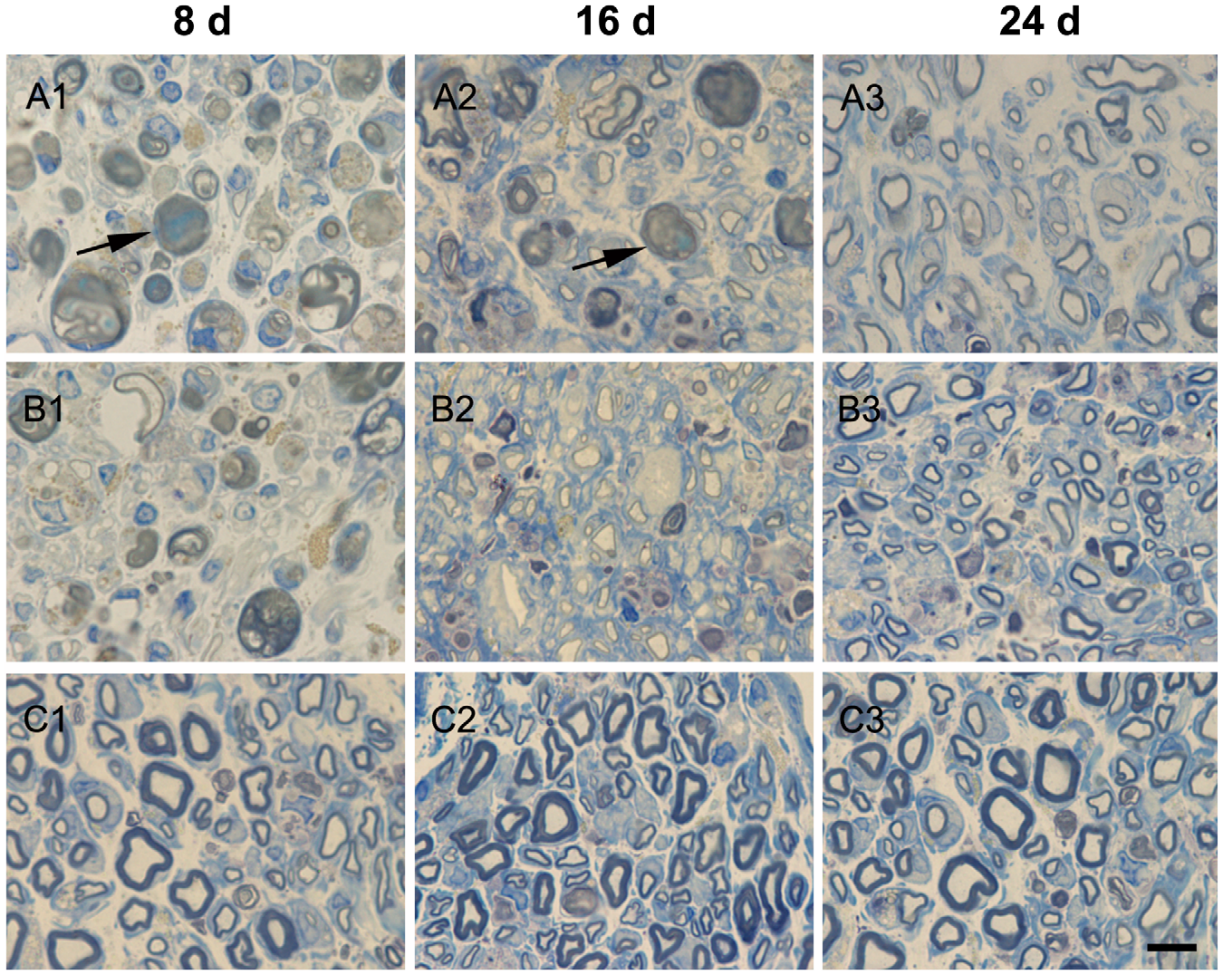
Semi-thin cross sections of sciatic nerve with toluidine blue staining. Panel A1, A2 and A3: The dystrophy appearance of nerve fibers after sciatic nerve-pinch injury at day 8, 16, and 24, respectively. The internal structure of the nerve was severely disorganized, sometimes with apparently enlarged nerve fibers (arrows) in Panel A1 or A2. Panel B1 and B2: The dystrophy appearance improved at day 8 and 16, respectively, after sciatic nerve-pinch injury with Gal treatment as compared with that in Panel A1 and A2. Panel B3: Most of the nerve fibers recovered to almost normal status at day 24 after sciatic nerve-pinch injury with Gal treatment. Panel C1, C2, and C3: Normal appearance of the sciatic nerve, with small and large diameter myelinated fibers regularly distributed in sham-operated control animals at day 8, 16 and day 24, respectively, after sham operation. Scale bar = 10 µm.

Most of the nerve fibers were undergoing some degree of dystrophy after sciatic nerve-pinch injury at day 8 and 16. The internal structure of the nerve was severely disorganized after sciatic nerve-pinch injury at day 8 and 16. Most of the nerve fibers showed recovery after sciatic nerve-pinch injury at day 24. The dystrophy appearance improved at day 8 and 16 after sciatic nerve-pinch injury with Gal treatment. Most of the nerve fibers recovered to almost normal status at day 24 after sciatic nerve-pinch injury with Gal treatment (Fig. 6).

### Ultrastructural alterations of sciatic nerve

**Figure 7:**
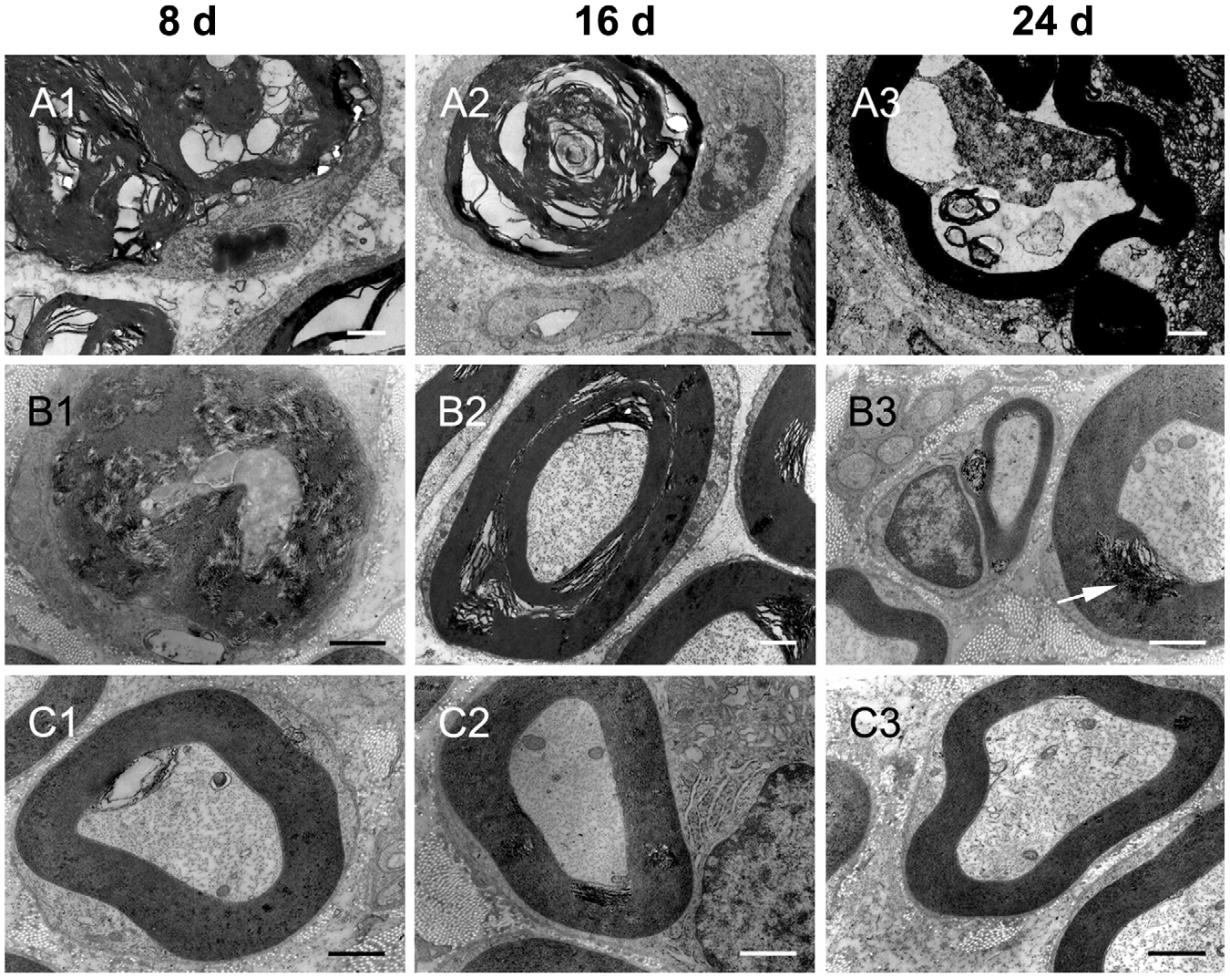
TEM micrograph of ultrathin cross sections of sciatic nerve. Panel A1, A2 and A3: Myelinated axons after sciatic nerve-pinch injury at day 8, 16, and 24, respectively. Degenerating myelinated axons are characterized by loss of axoplasm and subsequent myelin breakdown in Panel A1 and A2. Panel A3 showed the recovered myelin without clearly layers. The lost axoplasm is partially recovered. Panel B1, B2 and B3: Myelinated axons after sciatic nerve-pinch injury with Gal treatment at day 8, 16, and 24, respectively. The axoplasm loss and myelin breakdown in Panel B1 are less severe than those in Panel A1. The lost axoplasm is almost recovered and the broken myelin is recovering in most of the area in Panel B2. Panel B3 showed the recovered myelin with clearly layers. Only small areas have myelin debris (arrows) in Panel B3. Panel C1, C2, and C3: The ultrastructurally normal myelinated axons of sciatic nerve in sham-operated control animals at day 8, 16 and day 24, respectively, after sham operation. Scale bar = 1 µm.

Myelinated axons degenerated to myelin debris and lost axoplasm at day 8 and 16 after sciatic nerve-pinch injury. The myelin and axoplasm were partially recovered at day 24 after sciatic nerve-pinch injury. The axoplasm loss and myelin breakdown at day 8 and 16 after sciatic nerve-pinch injury were less severe in Gal treated animals as compared with that in sciatic nerve-pinched animals without Gal treatment. The broken myelin is recovering with clear layers and the lost axoplasm is recovered at day 24 after sciatic nerve-pinch injury (Fig. 7).

## Discussion

Treatment of chronic neuropathic pain resulted from peripheral nerve injury is one of the most difficult problems in modern clinical practice [25]. Current pharmacological treatments for neuropathic pain are not able to prevent or revert morphological and molecular consequences of tissue injury. On the other hand, many neurotrophins coexist restorative effects with hyperalgesia [1]. It has been shown that the neuropeptide Gal might have anti-nociceptive role in inflammatory conditions as well as the neurotrophic effects after nerve injury [17], [21]. In the present study, a dramatically upregulation of Gal in DRG and SDH caused by sciatic nerve-pinch injury was observed. Exogenous Gal administration plays an antinociceptive role in this nerve-pinch injury induced neuropathic pain animal model. It is very interesting that Gal, GalR1 and GalR2, which may play a key role in pain regulation and nerve regeneration, show great expression plasticity in DRG and SDH after nerve injury and intrathecal injection of exogenous Gal. Morphological investigation displays a serious damage in the pinched nerve and a facilitated regeneration after exogenous Gal treatment.

Mechanical injury of the sciatic nerve triggers numerous biochemical events in DRG and SDH, which respond to the neuropathic pain and nerve regeneration. A recent study with a sciatic nerve injury rat model indicated that one of the most conspicuous changes is Gal dramatically upregulated in DRG which will not return to normal until regeneration is complete [26]. It has, therefore, been suggested that Gal may be involved in the neuropathic pain regulation and nerve regeneration after peripheral nerve injury. It is well widespread accepted that sensory hypersensitivity followed by peripheral nerve injury is caused by the hyper C-fiber activity and sensitization of CNS, especially in SDH [27], [28]. During intrathecal injection, the administered drugs mainly diffuse directly into the superficial neurons in the SDH [29]. The superficial layers (laminae I and II) of SDH receives strong input from thin primary afferent fibers and is involved in nociception, pain, temperature sensing and other experiences [30], [31]. In the present study, intrathecal injection of exogenous Gal could ameliorate mechanical and thermal sensitization caused by sciatic nerve-pinch injury.

The observed antinociceptive effects and neuronal trophic effects of endogenous or exogenous Gal are likely mediated by the activation of GalR1 and GalR2 which are expressed in DRG neurons [32], [33]. It is also found a high level of GalR1, but not GalR2, expression in the interneurons of SDH. The levels of the both receptor subtypes display a great adjustability under difference situations [34]. Although the precise actions of Gal and its receptor subtypes in nociception and the exact sites of action have not yet been fully clarified, there is more and more evidence indicates that Gal could interact with the morphine/opioid receptor system and inhibit excited transmitter release to act as analgesia [35]–[37]. This effect is thought to be mainly mediated by GalR1 which is an inhibitory G-protein couple receptor. However, the GalR2, an excitatory G-protein couple receptor, is also proved to be a valid target in analgesia in CNS and PNS in several recent studies [36], [38]. Gal is expressed only at low levels in a small number of small-sized DRG neurons and mainly in lamina II neurons at normal condition and dramatically upregulates after nerve injury. Electrophysiology and behavior studies indicate that the physiological inhibitory role of Gal becomes more functional after peripheral nerve injury [15], [38]. This upregulation might be caused by lost connection between primary afferent neurons and target tissues. Disruption of axonal transport of neuronal trophic factors synthesized by peripheral target tissues removes the restrictive effect on Gal expression [39], [40]. These remarkable changes coincide with the finding that Gal acts as an important antinociceptive factor and neuronal trophic factor for injured neurons [41]. All the above researches indicate that Gal upregulation after peripheral nerve injury could be considered as a transform of normal neuronal sensory conduction state to a protection and regeneration one.

It has been demonstrated that nerve injury induces complex plasticity of Gal, GalR1 and GalR2 expression in DRG and SDH neurons, which play crucial role in neuropathic pain and nerve regeneration [26], [41]. In the present study, intrathecal injection of exogenous Gal caused a significantly downregulation of endogenous Gal in DRG. This change might be compensatory effect of exogenous Gal. Besides the dramatically change and important role of Gal after nerve injury, another very interesting question is whether and how Gal receptors change in this condition. In the present study, GalR1 expression was downregulated in DRG but upregulated in SDH, whereas GalR2 was upregulated in both DRG and SDH after sciatic nerve-pinch injury at the same experimental time point. Intrathecal injection of exogenous Gal decreased GalR1 levels but did not influence GalR2 expression, which coincide with our previous study in vitro [42]. The effect of depression of GalR1 by exogenous Gal may be an activator/receptor negative feedback just like the others receptor adaptive reaction. GalR1 has been confirmed to predominately locate in postsynaptic membrane [43]. Except inhibition of active neuropeptide release, activation of GalR1 may also reduce excitability of glutamatergic interneurons in SDH, thus cause antinociception [44]. The upregulation GalR1 in SDH makes this effect more potent after nerve injury, which may emphasize the important role of Gal in pain modulation in spinal level. In contrast to GalR1, which only couple to Gi protein, GalR2 can also activate Gq/11 protein leading to more complex functions [45]. Many studies pay close attention to neuroprotective and neuronal trophic effect of Gal conducted by GalR2 [46], [47]. In the present study, the GalR2 in DRG and SDH was upregulated, which makes the increasing Gal acts as neurotrophin more effective. An increasing and a relative independent expression of GalR2 in both DRG and SDH seem to be protected mechanisms, considerated its important role in nerve regeneration after nerve injury. Although it has been reported that GalR2 reduce its mRNA after axotomy [48], this difference may explain by the different model used here. Sciatic nerve axotomy is a severe damage, and even caused DRG neuronal death [49], but which can't be found in nerve-pinched animal model.

In the present study, neuronal trophic actions and nerve regeneration function of exogenous Gal were investigated by observation of MNCV, SNCV, toluidine blue staining and ultrastructural alterations after nerve-pinch injury. The recovery of NCV is responding to regeneration of motor and sensory fibers. Intrathecal injection of exogenous Gal significantly accelerated the functional recovery of limb as measured by MNCV and SNCV. The toluidine blue staining and TEM analysis showed more regenerative fibers in exogenous Gal treated animals as compared with that in rats without exogenous Gal administration after nerve-pinch injury. These morphological results further confirmed the tonic trophic effect of exogenous Gal after nerve injury.

In conclusion, Gal, via acivition of GalR1 and/or GalR2, may have neuoprotective effects in reducing neuropathic pain behaviors and improving nerve regeneration after nerve injury. These findings imply that interference with Gal and its recepotr system might be effective for neuropathic pain treatment. These data offer a new point of view on the treatment of neuropathic pain as well as the neuroprotective effects of Gal and its receptor signaling system.
